# Digital Workflows in Prosthodontics

**DOI:** 10.1111/adj.70005

**Published:** 2025-09-15

**Authors:** Jaafar Abduo, Vanya Rasaie

**Affiliations:** ^1^ Melbourne Dental School University of Melbourne Melbourne Victoria Australia; ^2^ Sydney Dental School University of Sydney New South Wales Australia

**Keywords:** 3D printing, AI dentistry, analogue, CAD/CAM, digital workflow, scanning

## Abstract

The rapid advancement of scanning technologies, design software, and manufacturing techniques has led to the maturation of digital workflows in prosthodontics over the past few decades. Initially perceived as alternatives to analogue workflows, digital workflows now offer significant advantages in diagnosis, treatment planning, communication, and prosthesis design and fabrication. The growing demand for efficient, predictable, aesthetic, and outcome‐driven prosthodontic treatments has led to increasing adoption of digital workflows. This integration has transformed each step of prosthodontic treatment, resulting in three digital workflows: laboratory, clinical, and combined clinical‐laboratory workflows. Although most prosthodontic treatments can now be executed digitally, a universally applicable digital workflow is yet to be established. Contrary to analogue workflows, digital workflows continue to evolve rapidly, with significant improvements anticipated in the near future that may establish them as the mainstream approach in prosthodontics. This review article aims to: (1) illustrate the importance of digital workflows in modern prosthodontics, (2) discuss available digital workflows in relation to different areas of prosthodontics, and (3) explore how recent advancements in digital dentistry are likely to shape the future of prosthodontics.

**Abbreviations**
3D: three‐dimensional
AI: artificial intelligence
CAD: computer‐aided design
CAM: computer‐aided manufacturing
IOS: intraoral scanning
ISB: implant scan body
MIP: maximum intercuspal position
VDP: virtual dental patient

**Summary**

- Digital workflows offer streamlined prosthodontic treatment from the diagnostic phase to treatment completion.
- In comparison to analogue prosthodontic workflows, digital workflows can facilitate more efficient diagnosis, controlled treatment progress, and more individualised prosthodontic treatment.
- Future digital workflow advancements are envisioned with the rapid technological developments.

## 1 Introduction

Prosthodontic treatment involves the replacement of missing teeth or parts of teeth, and oral tissue with a form of fixed or removable prostheses. The fundamental steps of any prosthodontic treatment are (1) diagnosis and planning, (2) recording the prosthesis supporting structures, (3) registration of the interarch relationship, (4) prosthesis design and (5) prosthesis manufacturing. Although analogue workflows have consistently demonstrated reliability in each of these stages, there has been a sustained interest in developing more efficient and predictable digital alternatives.

The journey towards digital prosthodontics began four decades ago with the introduction of the first intraoral optical impression and chairside design software and milling unit for fabricating simple single tooth indirect restorations [1, 2]. Although this attempt did not lead to a paradigm shift in prosthodontics workflow at that time, its innovative workflow sparked substantial interest and further development. Concurrently, computer‐aided design and computer‐aided manufacturing (CAD/CAM) emerged in the 1980s as an alternative manufacturing method, initially driven by rising noble metal costs [3]. This technology eventually enabled precise fabrication of metal prostheses [4] and became the sole reliable technique for fabricating prostheses from polycrystalline ceramics such as alumina and zirconia [5]. With the ongoing and rapid technological advancements, more efficient intraoral scanning (IOS) devices, powerful design software and reliable manufacturing hardware became a reality.

The integration of digital technologies has significantly transformed each stage of the prosthodontic treatment, leading to profound changes in treatment workflow. As a result, a variety of digital workflows are now available, offering enhanced predictability and efficiency [6, 7, 8, 9]. The adoption of digital workflows is further driven by the increasing demand for streamlined, cost‐effective and outcome‐oriented treatment approaches [6, 8, 9]. In many instances, digital workflows not only replicate but also surpass the capabilities of traditional analogue workflows, offering solutions that were previously unattainable [8, 9, 10, 11, 12, 13]. Unlike their analogue counterparts, digital workflows continue to evolve rapidly, with the potential for ongoing enhancements that outpace the current capacity of clinical and laboratory research to validate them scientifically [6, 8, 9]. This article aims to (1) highlight the importance of digital workflows in modern prosthodontics, (2) discuss the available digital workflows across various domains of prosthodontics and (3) explore how recent advancements in digital dentistry are likely to shape the future of digital workflows in prosthodontics. The article is intended to support clinicians in understanding the approaches to integrate digital workflows for an optimised prosthodontic treatment.

## 2 Significance of Digital Workflows in Prosthodontics

Although digital workflows still require a sound understanding of prosthodontic treatment by the involved clinicians and technicians, the integration of digital workflows within contemporary prosthodontics has evidently enhanced diagnosis and planning, recording prosthesis supporting structures, registration of interarch relationships, prosthesis design and prosthesis manufacturing. Although analogue workflows can still fulfil the criteria of acceptable prosthodontic treatment, digital workflows have substantially enhanced the delivery path. Specifically, incorporating digital technologies within prosthodontics has facilitated the following: (1) detailed diagnosis and treatment planning, (2) communication, (3) simpler treatment protocols and (4) enhanced prosthesis fabrication and materials.

### 2.1 Diagnosis and Treatment Planning

**FIGURE 1:**
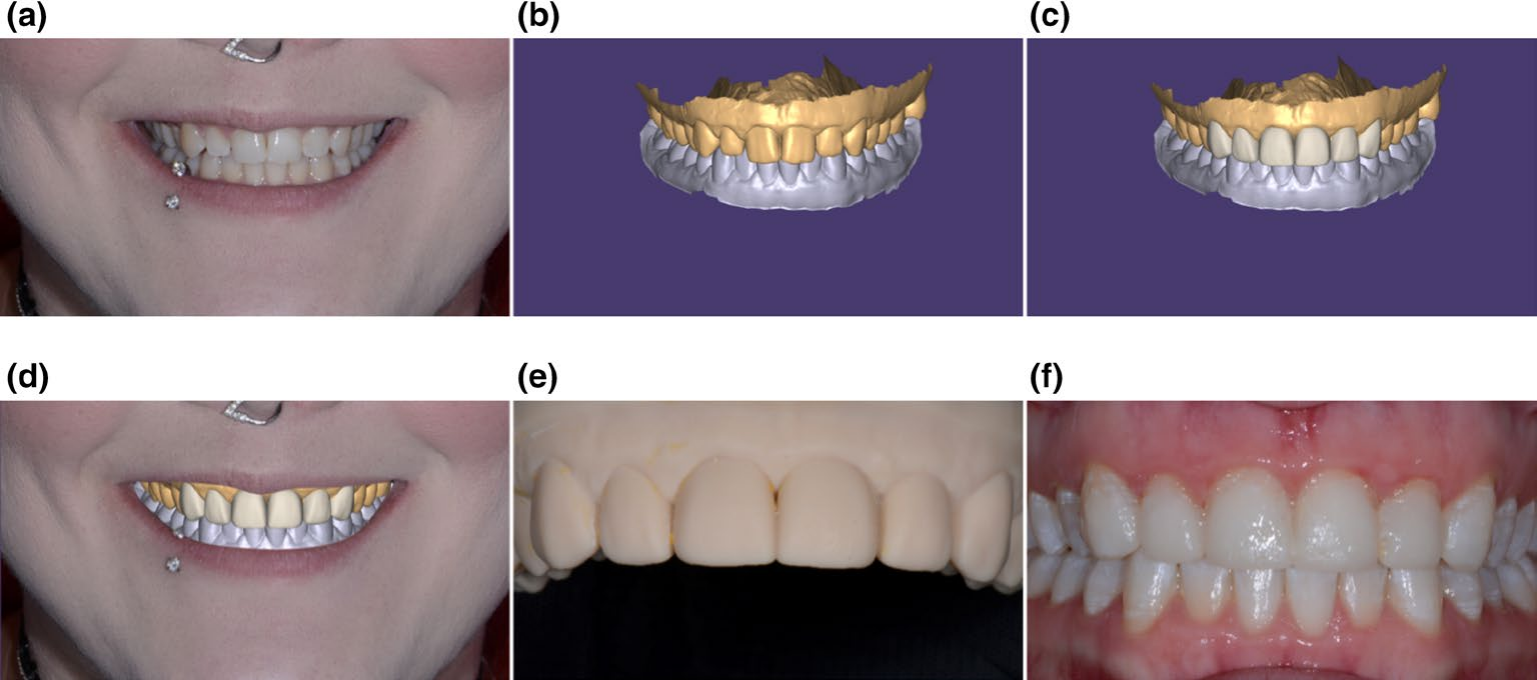
An example of the versatility of digital simulation of aesthetic treatment of maxillary anterior teeth. (a) Pre‐treatment frontal view. (b) Virtually articulated digital arches. (c) Digital treatment simulation involving the maxillary anterior teeth. (d) Instant visualisation of the digital wax‐up in relation to the facial profile. The digital simulation has the advantage of quick and reversible adjustments. (e) 3D printed model of the digital simulated treatment to enable intraoral translation of the intended plan. (f) Intraoral mock‐up to confirm the suitability of the digital simulated treatment prior to proceeding to the definitive treatment.

**FIGURE 2:**
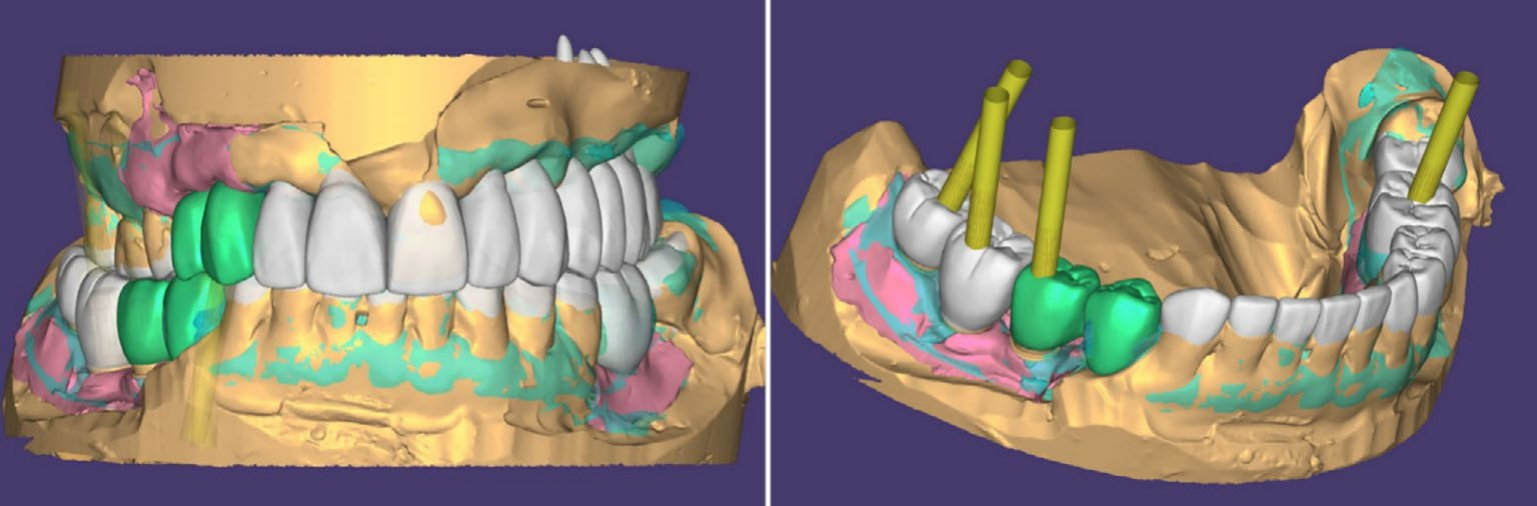
Digital treatment simulation can be reversibly conducted to evaluate the impact of treatment, assess multiple treatment options, and evaluate the impact of implant treatment.

**FIGURE 3:**
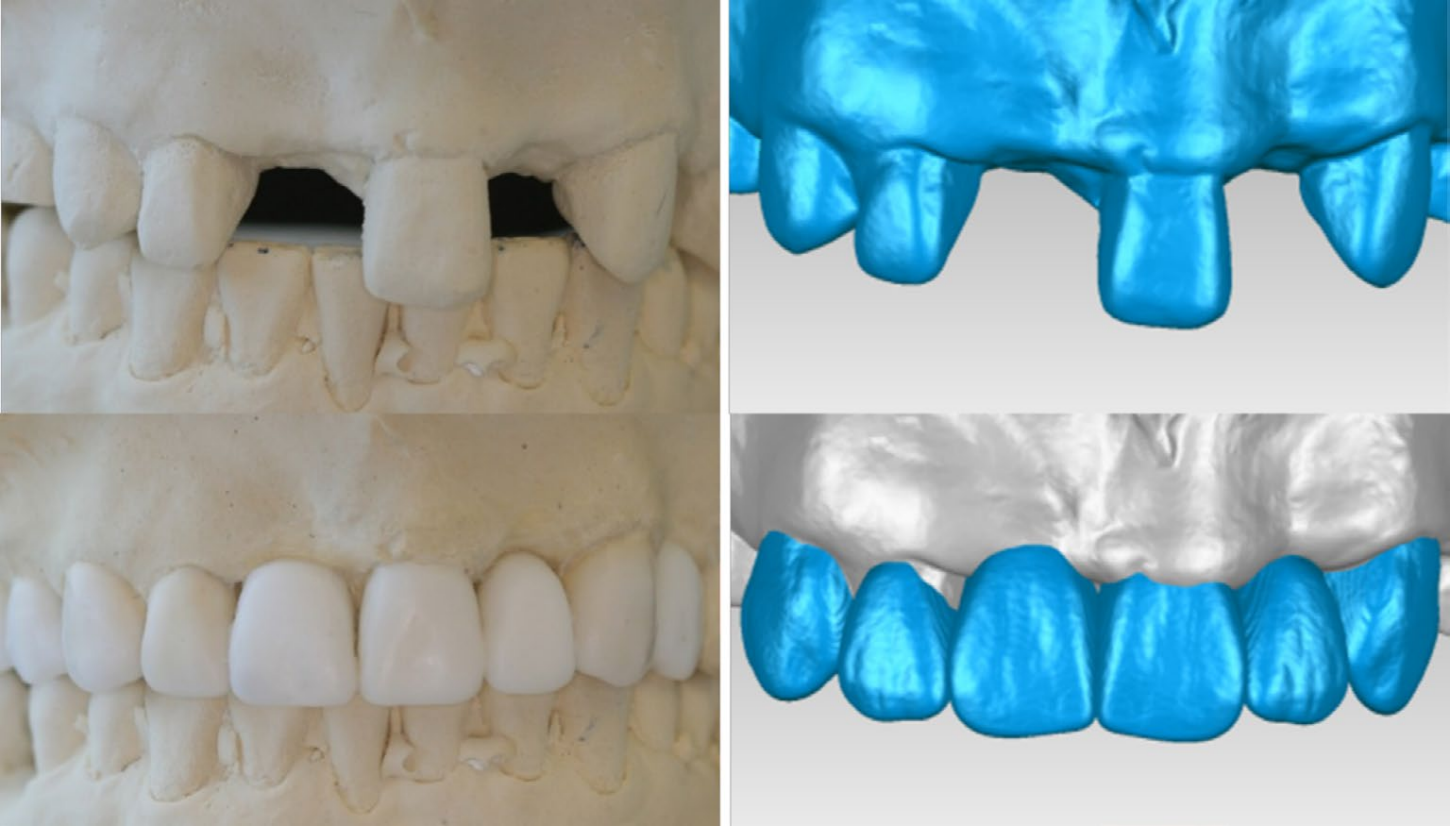
In comparison to analogue wax‐up, the digital treatment simulation has the advantages of detailed anatomical features, symmetry, and ease of adjustments.

The use of scanning technologies has enabled improved visualisation of the existing dentition, underlying structure and proposed treatment plans. Through digital planning software and their integrated teeth library, articulated virtual models can receive digital simulated treatment of the intended interventions (Figure 1). The CAD design features can further be individualised via the integration of digital smile design protocols and individual facial scan profiles. This enables a comprehensive visualisation of the aesthetic, functional and biological impact of the treatment on the remaining dentition. Preparation depth guidance template, non‐invasive testing of various treatments in a virtual environment and designing interim prostheses are some examples of facilitated assessment and visualisation that digital technologies can provide. Treatment involving dental implants, fixed prostheses, aesthetic restorations and removable prostheses can be reliably planned digitally (Figure 2). Compared with conventional analogue wax‐ups, digital treatment simulation offers substantial advantages, including enhanced efficiency, less time‐consuming and improved adaptability and reversibility [10, 11, 12]. The automated design features of the software will simplify treatment simulation, reducing dependency on technical waxing expertise. Several reports indicated that CAD software programs are capable of providing simulated treatment with superior aesthetics, symmetry and anatomical details compared with analogue wax‐ups that rely on the technician's skills (Figure 3) [10, 11, 12].

Several commercial software packages are available to execute multiple treatment simulations to enable flexible selection of the ideal treatment and identification of the impact of each treatment prior to treatment commencement. Eventually, judicious application of digital technologies for dental planning allows the treating clinician to select the most individualised treatment. Once the digital treatment simulation is approved by the patient and the treating clinician, it can be replicated precisely for designing and fabrication of the definitive prosthesis, which further reinforces predictable patient‐centred and outcome‐oriented treatment [7, 13, 14]. More recently, combining digital intraoral and extraoral virtual details with 3D radiographic images can establish a digital twin or the virtual dental patient (VDP), which facilitates a more accurate simulation of dental treatment. In addition, a prediction model of the simulated treatment can be performed and further supplemented with deep machine learning and artificial intelligence (AI).

### 2.2 Communication

**FIGURE 4:**
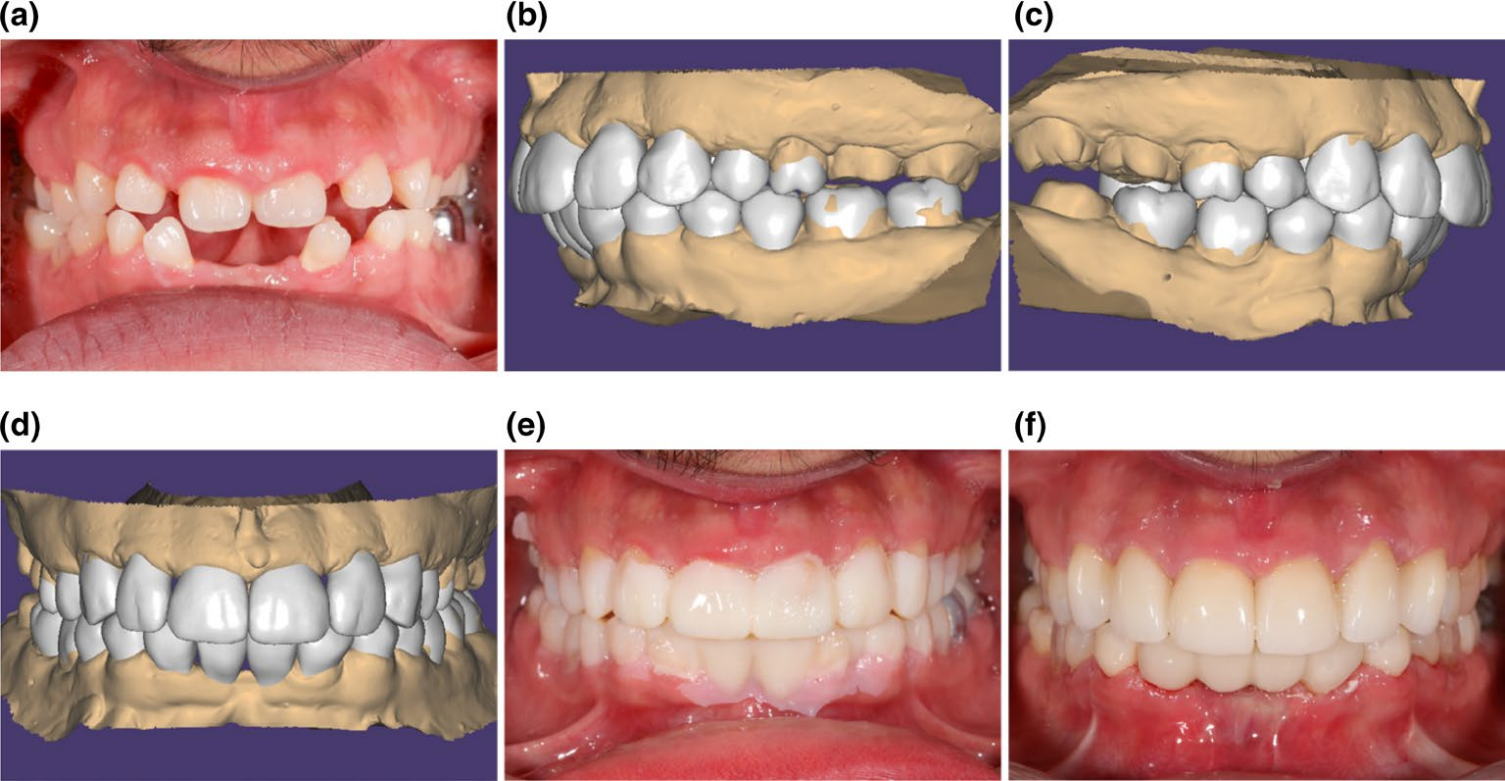
A clinical case illustrating streamlined digital workflow treatment from planning to treatment completion. (a) Pre‐treatment frontal view. (b–d) Completion of digital treatment simulation. (e) Intraoral mock‐up according to the digital simulation. (f) Once approved by the patient and the clinician, the virtual design can dictate the fabrication of the definitive prostheses (courtesy of Dr. Cindy Zhou).

Converting the patient presentation to virtual data has improved communication between the clinician and the patient, and the clinician and technician. The ease of digital data transfer to other team members, regardless of their location facilitates planning and controlled treatment execution. This further enhances communication among clinicians from different specialities. The intended treatment can be displayed to the patient digitally or applied intraorally according to 3D‐printed models. The design and manufacturing of prostheses can be carried out at separate locations, enhancing workflow consistency. Notable examples include 3D‐printed physical prototypes of the planned treatment, surgical guides and interim prostheses—all of which can be designed and fabricated remotely in specialised facilities or manufactured within the dental clinic. Eventually, streamlined communication within digital workflows enables a more cohesive progression from treatment planning to prosthesis delivery (Figure 4). Compared with analogue methods, digital workflows offer greater control over the definitive prosthesis design, enhancing precision and predictability throughout the process.

### 2.3 Simplified Treatment Workflow

**FIGURE 5:**
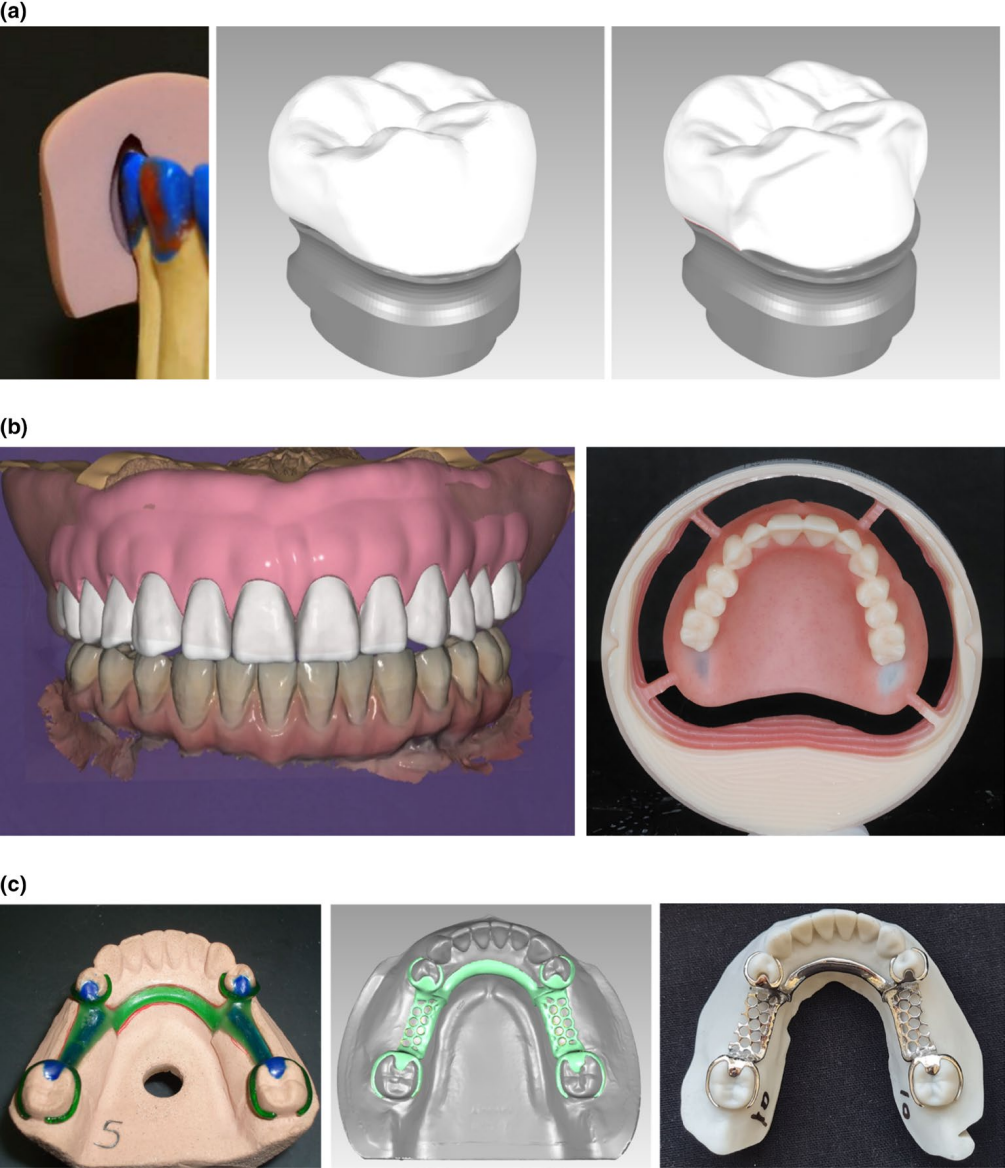
Examples of the simplification of laboratory procedures. (a) In comparison to analogue fabrication methods, digital design of prosthesis enables accurate and automated spacing and cut back. (b) Virtual teeth set‐up of complete denture in conjunction with milling substantially reduces the laboratory involvement time. (c) Contrary to analogue wax‐up of partial denture framework, which requires high technical skills, the digital design of the framework is integrated within several commercial software packages that allow for precise allocation of framework components. In addition, additive manufacturing eliminates the need for framework casting.

Digital workflows have demonstrated significant potential in reducing the number of treatment steps, as well as minimising both clinical and laboratory time across various prosthodontic procedures. In addition, IOS and digital prosthesis design were proven to be simpler and quicker as opposed to analogue workflows, with improved clinician and patient experience [8, 15, 16]. Furthermore, digital prosthesis design enabled high customisation of the prosthesis, such as digital cut‐back and pontic design. Further simplification is anticipated with the incorporation of automation features such as margin detection, virtual die space allocation, undercut identification, visualisation of the optimal path of insertion, pre‐designed teeth library selection, optimal teeth setup and validation of prosthesis dimensions (Figure 5a).

CAD software can also be reliably used for designing simplified removable prostheses. In removable prosthodontics, features like design automation, path of insertion visualisation, undercut determination and measurements, teeth setup and flange design (Figure 5b,c) have provided substantial benefit in simplifying the laboratory workflow. Automated teeth selection and setup contribute to a notable reduction in laboratory workload [8, 15, 16].

In implant rehabilitation, the utility of CAD software begins at the planning phase. Implant positioning can be determined on the basis of the desired prosthetic outcome, allowing for the design of surgical guides, evaluation of surgical invasiveness and, in certain protocols, the preoperative design of interim prostheses for immediate loading [17]. Furthermore, all the required components (e.g., healing abutments, definitive abutments and interim prosthesis design) can be determined prior to implant placement. In cases involving implant‐supported overdentures, digital planning enables precise estimation of attachment dimensions, contributing to the selection of optimal retention systems. All this is facilitated and simplified through the predictability that digital workflow provides.

### 2.4 Enhanced Prosthesis Fabrication and Materials

Digitally produced prostheses have been shown to have superior durability and accuracy. This has been attributed to fewer fabrication steps, reduced human intervention, reliable industrial‐grade manufacturing technologies and monolithic prosthesis design (Figure 5b) [9, 18, 19, 20]. The digital manufacturing techniques have facilitated the use of diverse materials, including reinforced polymers, base metal alloys, titanium and zirconia. Another advantage of the inclusive application of digital workflows is accurate patient‐specific prosthesis design, which contributes to enhanced prosthesis durability [21, 22, 23]. Clinical studies suggest modern manufacturing may reduce the incidence of some common mechanical complications observed by earlier studies on analogue workflows [24].

## 3 Digital Workflows

**FIGURE 6:**
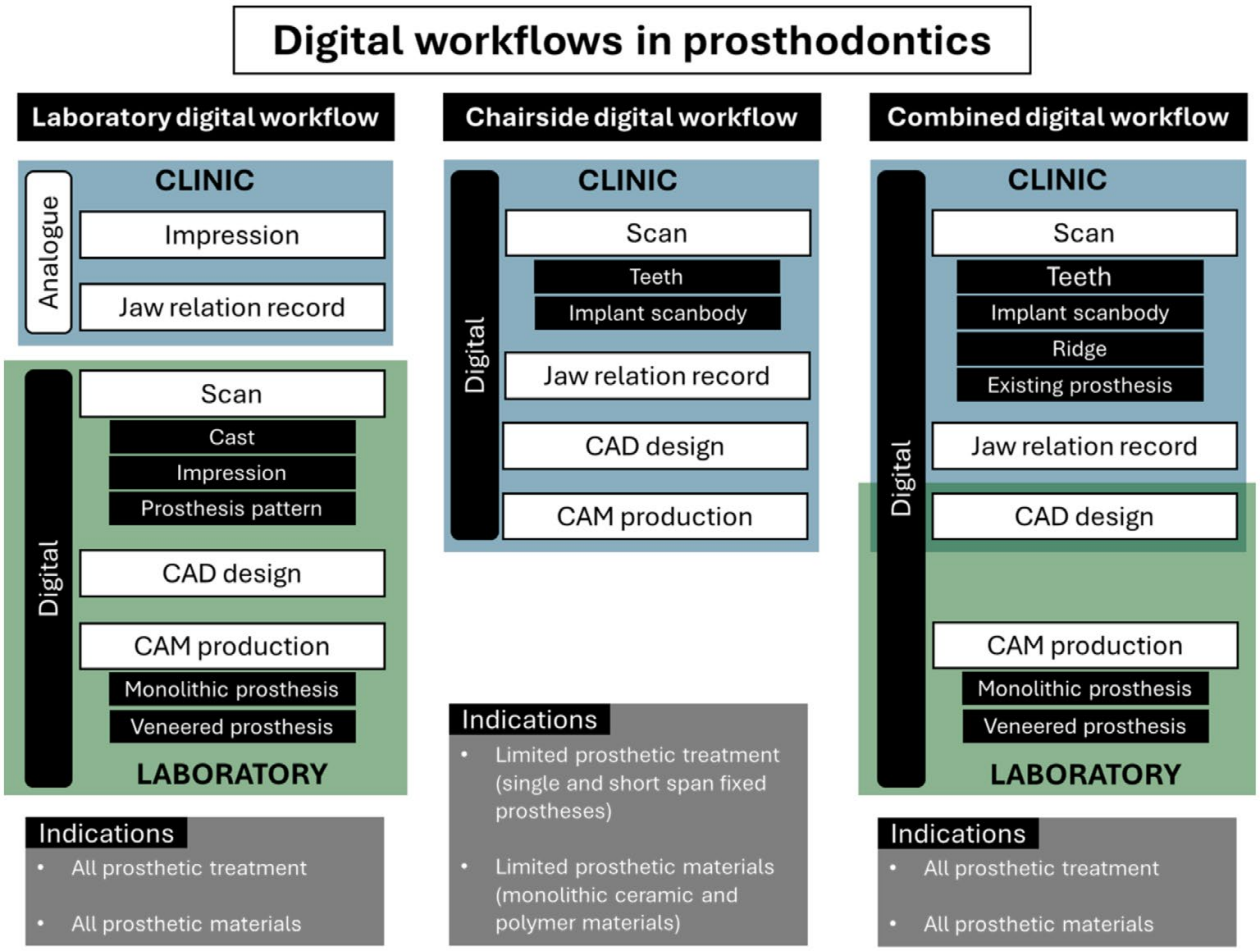
The available different digital workflows, with the different involvements of clinic and laboratory, and the indications of each workflow.

Prosthodontic treatment workflows comprise three integrated stages: data acquisition (impression and jaw relation record), prosthesis design and manufacturing. These three steps are available digitally through (1) the 3D acquisition with intraoral and/or extraoral scanners, (2) digital design with dental CAD software and (3) prosthesis manufacturing by a CAM system. These technologies are now accessible in both clinical settings and dental laboratories, and the digital workflow may be “complete workflow”, with the elimination of the dental laboratory role, or “incomplete workflow”, by relying on the dental laboratory. Currently, three prosthodontic digital workflows are available: (1) laboratory workflow, (2) clinical (chairside) workflow and (3) combined clinical‐laboratory workflow (Figure 6). The workflows vary in their capabilities, indications and procedural steps.

### 3.1 Laboratory Digital Workflow

Through this workflow, the clinician adheres to conventional clinical procedures, including standard diagnostic planning and work‐up, physical impression taking and registration of the inter‐arch and face‐bow records. The collected data are subsequently transferred to the laboratory through conventional means. The digital interaction occurs solely at the laboratory level, where the impressions or casts are indirectly digitised using a laboratory scanner [25]. Additionally, the models can be articulated via analogue means and scanned to be transferred into a virtual articulator. Several CAD software programmes include simulated virtual articulators on the basis of actual physical articulators [26]. The similarity between mechanical and virtual articulators in simulating dynamic movement and alteration of occlusal vertical dimension was found to be within 100 μm [27]. Subsequently, the prosthesis is designed and fabricated via CAD/CAM technology. Any wax‐up generated through analogue methods can be merged against the virtual working cast using the CAD software. Consistent with the analogue workflow, the prosthesis can be further modified and veneered by the dental technician. Additionally, the produced prostheses can be reevaluated on a mechanical articulator.

This incomplete digital workflow can be preferred by clinicians who are familiar with the traditional treatment workflow and prefer to utilise the advantages of digital manufacturing, such as precision, quality control and an increased range of materials. Further, this workflow enables the clinicians to execute modified conventional impression techniques (e.g., mucocompressive edentulous impression, detailed border moulding or splinted implant impression). Historically, this workflow has been shown to be more accurate than the workflows involving digital clinical steps. However, recent literature revealed consistent improvements in clinical digital steps and, in several aspects, exceeded the outcome of analogue clinical steps [28, 29]. The digital workflow at the laboratory level typically necessitates a comparable number of clinical visits to that of the analogue workflow and may retain several inherent limitations associated with traditional techniques. For instance, distortion of conventional impressions, the necessity of impression disinfection and dimensional changes of the stone cast [30]. The drawback of this workflow is the loss of clinical advantages and the convenience of all digital dentistry tools. The laboratory digital workflow is applicable for all prosthodontic treatments and can utilise all prosthetic materials.

### 3.2 Clinical (Chairside) Digital Workflow

This workflow is a complete digital workflow, where all clinical and technical steps are completed digitally by the clinician at the dental office. This requires the use of an IOS unit and a chairside CAD/CAM unit (3D printer or milling machine). The clinician is responsible for the prosthesis design. Multiple clinical steps are combined in a single clinical visit, which substantially reduces treatment duration and patient inconvenience. The effectiveness of this approach is largely contingent upon the clinical armamentarium and clinician's ability to provide adjunctive procedures such as sintering, staining and glazing. Despite its effectiveness, the workflow is subject to notable limitations, primarily because of the constrained capabilities of chairside additive and subtractive manufacturing systems. For example, only single‐unit and short‐span prostheses can be produced because of the small dimensions of the milling block. In addition, the prosthesis can only be produced from monolithic ceramic or reinforced polymer, which cannot be modified by veneering. Although single‐unit and short‐span implant prostheses can be produced, they are restricted to ceramic or reinforced polymer materials on the prefabricated metal insert available at the software library [31]. Once fabricated, the implant prosthesis is cemented onto the prefabricated abutment either intraorally or extraorally. However, this approach may restrict the clinician's ability to modify the peri‐implant soft tissue contour and preclude the fabrication of custom abutments. Although 3D printing enables the production of larger‐span prostheses, the limited mechanical durability of current printable materials renders these restorations suitable primarily for interim use [32]. Further, the level of artistic customization of the prosthesis is minimal because of the lack of technician involvement. At present, removable prostheses cannot be fabricated within this workflow. Nevertheless, ongoing advancements in additive manufacturing technologies and material science are expected to broaden the scope and application of this approach.

### 3.3 Combined Clinical Laboratory Workflow

This workflow is characterised by digital execution of the clinical procedures within the dental clinic and fabrication of the prosthesis by a commercial dental laboratory or manufacturing centre. As all the data and prostheses can be generated digitally, it is a complete digital workflow. Once the IOS is obtained by the clinician, the virtual image is imported into planning software, where the prosthesis design can be completed either by the clinician or the technician. In addition, this workflow can be implemented at the treatment planning phase, where the dentition is scanned and transferred to a CAD software for digital treatment simulation that can later be generated by 3D printing. The increasing popularity of this workflow is attributed to the efficacy of virtual data transfer and the combined advantages of the two other described workflows [33]. Involvement of the dental technician enables enhanced aesthetic customization and veneering. To improve the outcome of this workflow, it is advised to generate a 3D printed dental cast on which the prosthesis can be seated and adjusted in the laboratory setting. Although this workflow can be used for all prosthodontic treatment and materials, it may require additional considerations for removable prostheses and long span implant prostheses to overcome the limitations of the IOS of long span scanning and recording of soft tissue. The validity of this workflow is highly dependent on:

- IOS, and
- Digital jaw relation recording and virtual articulation.

#### 3.3.1 IOS

Several factors were proven to impact the accuracy of IOS. The most critical factors are the span of IOS and the scanned surface [34, 35, 36]. Other factors involve the operator's experience, ambient temperature [34], ambient light illuminance [37], scanning distance, angle and scanning pattern [38, 39]. Patient‐related factors such as existing restorative material, level of humidity, misaligned teeth, dental arch width, tooth type and tooth colour may also affect the quality of IOS [39, 40]. Of interest to this workflow, the different scanned surfaces (soft tissue, teeth or implants) require different considerations.

##### 3.3.1.1 Soft Tissue Scanning

Most modern IOS units have shown similar accuracy to conventional impression techniques in capturing the attached mucosa on the alveolar ridge and palatal areas, with accuracy considered acceptable within the threshold values for fabrication of conventional removable dentures (300 to 500 μm). However, IOS units struggle to accurately capture the mobile tissue in vestibular areas and the floor of the mouth [36]. This limitation prevents IOS from being used as definitive impressions in the digital workflow for complete denture fabrication, making indirect digitisation by laboratory scanner of the cast or IOS of conventional impression a more reliable approach [41, 42]. Technique modifications have been described to mitigate the challenges of scanning an edentulous mucosa, including the addition of artificial markers to the long‐span edentulous area [43], and following a specific scanning strategy [39]. In partial denture cases with substantial edentulous areas, IOS is sufficient as a primary impression method. Definitive conventional impression approach may become more critical when functional or selective pressure techniques are needed for optimal clinical outcomes [36, 44]. If the partial denture is primarily tooth‐supported with confined edentulous areas, the IOS as a definitive impression is a reliable approach [45].

##### 3.3.1.2 Teeth Scanning

Fully dentate arches in prosthodontics are scanned to capture the intact dentition or the prepared teeth. Several studies showed that digital impressions provide similar or slightly better accuracy than polyether or polyvinyl siloxane impressions in partial arch cases [35]. Analysis of the marginal and internal fit of restorations made via IOS confirmed its reliability for producing single crowns and short‐span fixed dental prostheses [30, 40, 46]. The accuracy of IOS is further optimised with marginal visibility and access to proximal surfaces. As for full‐arch impressions, mixed results have been shown in terms of accuracy when comparing IOS to conventional impressions, with most studies favouring conventional impressions over full‐arch scans [35]. Contrary to short‐span scanning, where the stitching errors accumulate over only a short distance, long‐span scanning may experience substantial errors accumulation over the length of the arch. Nevertheless, as tooth‐borne prostheses for the full‐arch are fabricated separately as single units or short‐span prostheses, errors of full‐arch scanning may influence the interproximal contact quality, and the fit on the prepared teeth is less likely to be affected. However, the clinical significance of this finding is yet to be determined, as the cementation space may compensate for the minor IOS errors. For diagnostic scanning, a consistent finding is that IOS and alginate impressions offer similar accuracy and can be used alternatively [35, 47, 48, 49]. Therefore, given the lack of consensus on the required accuracy for clinical full‐arch scans, these findings should be interpreted on the basis of the specific goals of the scan [48].

##### 3.3.1.3 Implant Scanning

**FIGURE 7:**
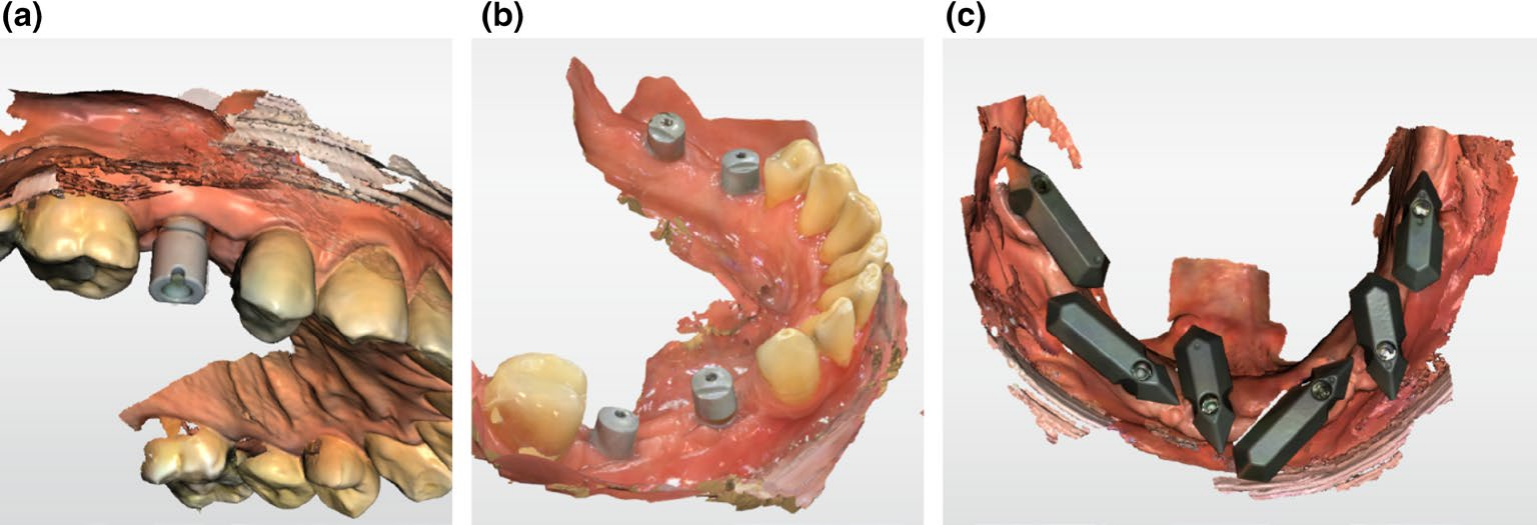
Examples of different applications of ISB. (a, b) ISB for single implant scanning and short span multiple implant scanning can easily be accessed and scanned and is less affected by mobile soft tissue. (c) Full‐arch implant scanning with modified ISB to avoid the reliance on soft tissue.

IOS is steadily increasing for fabricating restorations on single implants, multiple adjacent implants and short‐span implant fixed dental prostheses (Figure 7a,b) [50]. Although implant location, depth, alignment and implant scan body (ISB) design (material, size and geometry) may affect the IOS accuracy [51, 52], IOS was consistently shown to provide reliable clinical accuracy compared with conventional impression techniques [9, 50, 53], which makes IOS a substitute for conventional impression. Scanning implants is thought to be more reliable than teeth because it relies on capturing parametric surfaces of the ISB and further converting them to the parametric morphology of the implant connection or abutment surface. The clinician has the flexibility of selecting ISB suitable for the different clinical presentations. In addition, IOS of implants appears to be less affected by the implant angulation as no material distortion is involved [51].

Contrary to single and short span fixed implant prostheses, open‐tray splinted conventional impression is still considered the gold standard for full‐arch implant rehabilitations [43]. This is mainly because of the presence of soft tissue surface between implants, especially in cases with a lack of keratinized mucosa or a severely resorbed alveolar ridge. This can hinder proper stitching or cause misinterpretation of the scanned data [36]. To mitigate this limitation, several scanning methods have been proposed [54, 55, 56, 57, 58]. Revilla‐Leon et al. have classified them into four categories: non‐calibrated splinting of ISBs, calibrated ISBs, calibrated framework technique and reverse impression technique of existing prosthesis [59]. An auxiliary device, such as a printed arch‐shape plate or bar with teeth or anchor pins between the ISB, and soft tissue markers between the ISB are examples of non‐calibrated splinting techniques [54, 55, 56]. The concept is to fill the inter‐implant distance with geometrically complex features and reduce the dependence on soft tissue. Additionally, teeth on the devices can be used for registering the inter‐arch relationship [58]. The literature is unequivocal on the optimum non‐calibrated splinting technique. Thus, although these techniques could potentially allow for a complete digital workflow, a verification jig is recommended when IOS is applied with non‐calibrated splinting techniques [58, 59]. Specifically calibrated ISBs with auxiliary features and scan gauges to enhance the scan body geometry and reduce the stitching errors have been developed (Figure 7c). Some of the commercially available systems are Nexus iOS (Osteon), IPG (Shining 3D) and ioConnect (TruAbutment) [60]. Currently, the available short‐term clinical data support the promising outcome of these modified ISB for full‐arch implant rehabilitation [61]. Papasprydakos et al. investigated the reverse scan body technique (RevEX, Straumann), which involves attaching scan bodies on the fitting surface of the interim prosthesis for extraoral scanning with an IOS [58]. The concept simulates the back‐pouring technique in a digital workflow. However, such advanced scanning strategies may restrict the operator in selecting a specific manufacturing centre, implant components or even an IOS system.

Photogrammetry (e.g., iCam4D and iMetric4D), which creates 3D models from extraoral scanning of coded ISB, offers an alternative to IOS in digitising the implant location and angulation. The advantage of photogrammetry is the large view of scanning that captures all the ISB simultaneously. Clinical and laboratory studies investigating photogrammetry systems have shown accurate results independent of the number of implants, faster scanning and higher patient and operator satisfaction [62, 63, 64]. Although the photogrammetry systems are accurate in capturing the 3D implant position, they cannot capture adjacent teeth or soft tissue, requiring supplemental IOS of the soft tissue to determine prosthesis adaptation. This limitation, along with high equipment costs, has restricted its widespread use.

#### 3.3.2 Digital Jaw Relation Recording and Virtual Articulation

Through IOS, the occlusal relation is obtained directly via the integrated buccal scans of the dentition in occlusion. For recording articulation at maximum intercuspal position (MIP), a virtual articulator has been confirmed to be reliable [65, 66]. For a minimal number of units in a single quadrant, reliable articulation can be achieved with single side scanning, and IOS have demonstrated superior accuracy compared with analogue methods when recording static MIP in quadrant dentistry [67]. However, for complex rehabilitation involving long span prostheses, two sides scanning is necessary. Recent studies assessing the accuracy of full‐arch MIP registration using IOS have reported performance comparable to conventional impression methods [68, 69]. However, such comparable results have been noted primarily for completely dentate arches, arches with a single missing posterior tooth, stable occlusion and a single prepared tooth [69]. Additional factors influencing the accuracy of IOS in recording static MIP include the size and location of occlusal contacts, the position and size of edentulous areas and the specific IOS technology employed [67, 69].

For several clinical presentations, a stable MIP does not exist and requires establishment prior to IOS recording. This can be observed in situations of minimal natural tooth contacts, the necessity of altering occlusal vertical dimension, long‐span and full‐arch implant prostheses and completely edentulous arches. Achieving occlusal stability is essential to complete this workflow. This can be in the form of using an interim prosthesis or occlusal registration rim. Once the new occlusal relation is established intraorally, direct scanning can be obtained via buccal scans or indirect scanning of the occlusal record [68].

The complete digital workflow follows a modeless workflow and entails the use of virtual articulators that are incorporated with most dental CAD systems. In addition, some technologies are available to approximate the dynamic occlusion recording in a complete digital workflow, including virtual motion simulation, jaw tracking systems and computerised occlusal analysis systems. However, only some of these systems offer the ability to integrate the recorded data into the CAD software programmes for designing dental prostheses. A study examining the accuracy of IOS‐integrated motion trajectories concluded that the current technological capabilities may not reliably replicate dynamic occlusal records during eccentric mandibular movements [70]. Another digital technique for registering the interarch relationship is the jaw tracking systems to record mandibular motion during excursion and mastication. Although the data derived from these systems facilitate the complete digital approach for the occlusal analysis and adjustments, their accuracy is yet to be determined [71]. Further studies are required to assess the accuracy and efficacy of these systems in comparison to analogue methods. To date, virtual simulation of dynamic motion without a physical articulator is still experimental and the accuracy is yet to be determined.

In an attempt to increase the accuracy of simulated articulation, a virtual facebow has been integrated with the articulation [72, 73]. A feasible virtual facebow recording can be achieved through relating the arches to the temporomandibular joint via a large field cone beam CT [72]. However, the large field of exposure might not be required or justifiable in all clinical situations. Extraoral scan body systems have been introduced to integrate the facial scan data with the virtual maxilla [73, 74, 75]. A simpler approach has been suggested to rely on 2D photographs to capture the horizontal plane in relation to reference glasses [76, 77]. However, virtual facebow recording techniques are still experimental, and their clinical benefits and accuracy are yet to be determined [75, 76, 78].

## 4 Future Directions

**FIGURE 8:**
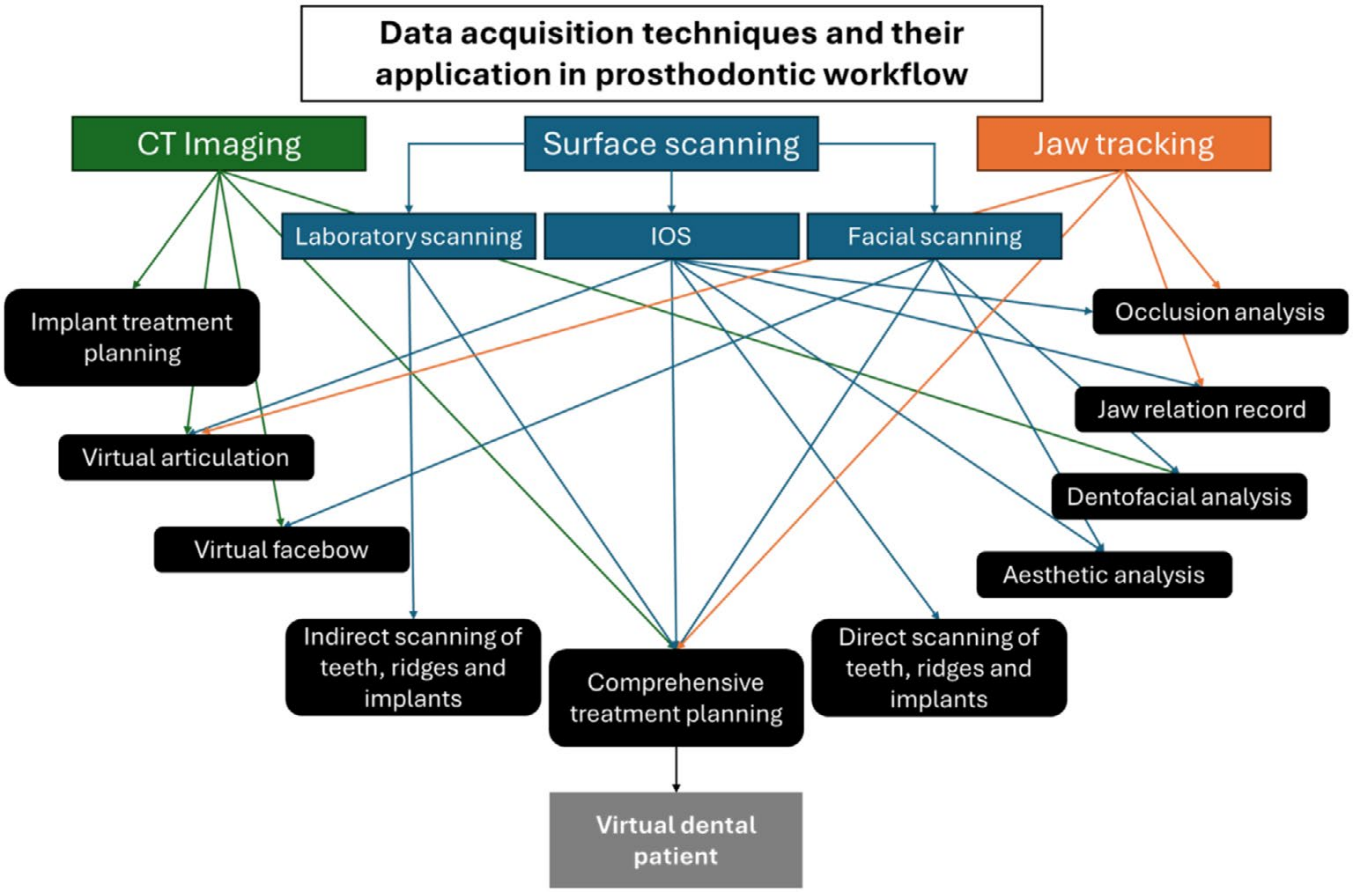
Schematic representation of the different data acquisition techniques and their interrelationship in generating comprehensive virtual data of individual patients. Eventually, VDP can be produced to accurately simulate the patient prior to the treatment.

With ongoing and progressive advancements, it is anticipated that further significant developments will emerge in the near future. Although some of these technologies are currently available, the clinical impact of their application remains to be fully established. This includes the incorporation of facial scanning, virtual dental patients and AI‐integrated treatment approaches (Figure 8).

### 4.1 Extraoral Scanning (Facial Scan)

Facial scanners are commercially available as stationary and handheld devices, and they are based on stereophotogrammetry, laser‐beam, or structured light projection technologies. Stationary stereophotogrammetry scanners record all the facial morphology simultaneously by one click operation, which reduces the operator errors and scanning issues. As a result, they have demonstrated superior geometric trueness and precision [79]. However, their high economic investment and complex scanning and calibration protocol have hindered their widespread adoption [80]. In contrast, handheld scanners capture the surface morphology by manually moving the device around the patient's face to build the 3D face image. The prolonged scanning time can introduce operator's error to the scan. Additionally, it might be challenging for the patient to hold the same smile (maintain the same posture) for more than 5 to 10 s. However, handheld scanners with structured light technology are a simpler and more affordable alternative [81]. This technology has been further incorporated into smartphones and tablets that are under the umbrella of the handheld scanners.

Studies that evaluated the accuracy of different facial scanner technologies have shown deviation values close to 1 mm. However, 2 mm deviation values have been considered to be clinically acceptable [75, 82]. The accuracy of mobile device‐based facial scanners is reported to be within the acceptable range, which makes them suitable for the clinical application of diagnosis and treatment planning [80].

### 4.2 VDP

Today, it is possible to combine all the virtual data to generate the VDP as a digital twin of the actual patient [7, 83]. These data may include IOS, extraoral scanning, 3D radiographs, and recording of dynamic mandibular movement. VDP offers visualisation of the merged data and facilitates the process of planning and prosthesis design. The ultimate aim is generating a treatment plan and prosthesis design that match the patient's dentofacial profile for optimum predictability, comfort, aesthetic and function [14]. VDP is likely to be more common in the future, and is thought to facilitate predictable patient‐centred treatment, outcome‐driven treatment, interdisciplinary treatment planning and clearer communication with the patient and other treatment team members. In addition, the planned treatment of the individual patient can be functionally simulated to determine its likely performance.

### 4.3 AI‐Integrated Prosthodontics

The integration of AI and automated tools could potentially enhance usability and contribute to user‐friendliness and a reduced learning curve of digital workflows. All the CAD design features, such as margin outlining, occlusion design and prosthesis dimension determination, can be augmented with AI integration [84], and it is anticipated that AI will play a greater role in CAD optimisation in the near future. The available current literature indicates promising outcomes in accurate shade selection, ideal aesthetic determination, automated tooth anatomy design and the design of removable prostheses [84]. It is envisaged that in the future, AI will play a greater role in automated diagnosis and treatment planning, and more advanced prosthesis design. This may involve patient‐centred and individualised treatment planning, comprehensive prediction models of facial changes and long‐term prosthesis performance [85]. However, the lack of a unified consensus among experts has made it difficult to create AI models that align consistently with the broader scientific understanding [86]. This inconsistency highlights the need for standardised guidelines to support the development of reliable and universally applicable AI‐driven design tools. Despite the promising contribution of AI in prosthodontics, more studies are needed to confirm its validity in different aspects of prosthodontics [86, 87].

Although AI‐integrated technologies provide significant support to dental professionals, it is important to remember that they should not replace the critical thinking and specialised knowledge that human practitioners contribute to patient care. Overreliance on AI may lead to a passive approach, where dental experts accept AI‐generated evaluations without carefully analysing them. This becomes especially concerning in complex cases where AI might not fully grasp the complexity of the individualised patient's condition. Therefore, it is essential to develop robust regulatory systems to guide the use and integration of AI in dental practice. These frameworks should guarantee that AI tools are not only reliable and effective but also used ethically, protecting patients from potential risks and ensuring the highest standards of treatment are maintained.

## 5 Summary

Contrary to analogue workflows, digital technologies in prosthodontics offer streamlined workflows from the diagnostic phase to treatment completion. Digital workflows offer substantial advantages over analogue workflows in terms of efficient diagnosis, controlled treatment progress and more individualised prosthodontic treatment. Because of the rapid technological advancements and AI integration, additional improvements in digital workflows are likely to occur in the future. Although a consistent digital workflow cannot yet be generated for each prosthodontic treatment, a form of complete digital workflow will likely be the streamlined approach for future prosthodontic treatment.

## Author Contributions

**Jaafar Abduo:** conceptualisation; writing – original draft; and writing – review and editing. **Vanya Rasaie:** conceptualisation; writing – original draft; and writing – review and editing.

## Conflicts of Interest

The authors declare no conflicts of interest.

## Data Availability

The data that support the findings of this study are available from the corresponding author upon reasonable request.
